# Prioritizing Mental Health within HIV and Tuberculosis Services in PEPFAR

**DOI:** 10.3201/eid3004.231726

**Published:** 2024-04

**Authors:** Rena Fukunaga, Paul Pierre, John K. Williams, Rebeca Briceno-Robaugh, Sam Kalibala, Meaghan Peterson, Patrick K. Moonan

**Affiliations:** Centers for Disease Control and Prevention, Atlanta, Georgia, USA (R. Fukunaga, J.K. Williams, P.K. Moonan);; US Agency for International Development, Washington, DC, USA (P. Pierre, R. Briceno-Robaugh, M. Peterson);; Office of the US Global AIDS Coordinator and Health Diplomacy, Washington (P. Pierre, S. Kalibala)

**Keywords:** Mental health, PEPFAR, HIV/AIDS, tuberculosis, substance use disorders, United States

## Abstract

Underprioritization of mental health is a global problem and threatens the decades-long progress of the US President’s Emergency Plan for AIDS Relief (PEPFAR) program. In recent years, mental health has become globally recognized as a part of universal healthcare, making this an opportune moment for the global community to integrate mental health services into routine programming. PEPFAR is well positioned to lead by example. We conceptualized 5 key strategies that might help serve as a framework to support mental health programming as part of PEPFAR’s current 5-year strategic plan. PEPFAR and the global community have an opportunity to identify mental health service gaps and interweave global mental health priorities with actions to end the HIV and TB epidemics by 2030.

Confronting the global burden of mental health disorders is a necessary measure to end the dual epidemics of HIV and tuberculosis (TB). HIV, TB, and mental disorders, including those tied to substance use, are closely interwoven (*1*). Persons with HIV and TB are at greater risk of experiencing mental illness compared with the general population (*2*,*3*). Close to 1 million TB cases (*4*) and as many as 33,000 HIV-related deaths are attributed to alcohol use disorders each year worldwide (*5*). Mental health conditions exacerbate poor healthcare behaviors and outcomes across both the HIV care continuum (*6*) and the TB treatment cascade (*7*) (e.g., seeking care, missed treatments, loss to follow-up), further accelerating both epidemics. Of note, COVID-19 has disrupted both public health and psychiatric service delivery and further widened the mental health treatment gap for those who need it most (*8*). Mental illness, HIV, and TB cannot be siloed; basic strategies aimed to eliminate HIV and TB rely on a unified response that is integrated with mental and behavioral healthcare services (*9*,*10*).

Underprioritization of mental health issues is a global problem (*11*). The 2022 World Mental Health Report found the treatment gap for severe forms of mental health conditions to be at a staggering 90% (*12*). National health systems, on average, allocate only 2% of their budgets each year to preventing and treating mental health conditions; however, in most low- and middle-income countries, the average allocation is considerably less (0.5%) (*12*). In addition to scant spending, many countries face substantial scarcities in trained personnel dedicated to mental health. There remains a dire workforce shortage in low-to-middle-income countries, which average <1 mental health worker per 100,000 population (*12*). Moreover, the limited availability of essential psychotropic drugs poses a significant barrier for accessing mental health treatments in those countries because of inequities in the pricing, availability, and listing of essential psychotropic medicines (*12*). Thus, mental health coverage and availability of services remains out of reach for most persons globally, including children and adolescents, for whom mental health services are known to be almost nonexistent (*12*).

In 2013, the World Health Organization (WHO) Comprehensive Mental Health Action Plan 2013–2020 was enacted and provided the first framework for countries to implement their own mental health policies and programs (*13*); this plan was subsequently updated in 2021 (*14*). In 2019, the first United Nations high-level meeting on Universal Health Coverage (UHC) recognized mental health as a core component of UHC. To achieve the goal of UHC across the world, member states acknowledged the need for mental health programs to be integrated with service delivery at the community level and be covered under financial protection arrangements (*15*). In the same year, the US President’s Emergency Plan for AIDS Relief (PEPFAR) provided guidance on the importance of integrating mental health services into HIV programs across the prevention, care, and treatment cascade (*16*). Most recently, the new 5-year strategy set forth by the Global Fund to Fight AIDS, Tuberculosis, and Malaria—The Global Fund Strategy (2023–2028) —recognized, for the first time, the key role mental health services play in accelerating efforts to end HIV and TB (*17*). Of note, key global partners, such as WHO (*18*), the World Bank (*19*), the Joint United Nations Programme on HIV/AIDS (*20*), the International Union Against Tuberculosis and Lung Disease (*21*), and the Bill & Melinda Gates Foundation (*22*), have also highlighted, through their own strategic priorities and program activities, the importance of addressing the comorbidity of HIV, TB, and mental health disorders. Such commitments made by stakeholders have marked a historic moment for mental health integration in HIV and TB programs. Yet, there has been minimal inclusion of mental health programming in both the United Nations’ Millennium Development Goals and Sustainable Development Goals. To date, specific policies and programmatic practice concerning mental health are not fully implemented by most countries (*12*), and it is conceivable that some interventions planned in high-resource communities may not be practical to implement elsewhere without substantial investment and additional human resources. Chronic underinvestment in mental health infrastructure, services, and research in low-income and middle-income countries remains a key reason for poor access to mental health services, as well as availability and reporting of mental health data (*23*). Efforts to scale up mental health programming should consider a health systems framework that ensures mental health services are seamlessly woven into treatment options across national disease programs.

PEPFAR is well-positioned to lead the way by strengthening the health system and supporting mental health treatment and services for persons living with or at risk for exposure to HIV and TB. Efforts have already been put in motion to integrate psychosocial support interventions into the work carried out by PEPFAR-supported staff (*24*). However, direct specialized mental health and addiction services have not been widely adopted by countries in which those staffs operate. PEPFAR-supported countries are encouraged to coordinate and collaborate with national behavioral health programs and services supported by other funders to accelerate the integration of HIV, TB, and mental health services (*25*). PEPFAR has decades of experience and well-documented flexibility and capacity to adapt strategies based on the country’s availability of health and human resources (*26*). This capacity might be also applied to the integration and implementation of mental health services at the community levels.

Given the increasing momentum to recognize mental health as a part of UHC globally, now is an opportune moment to strengthen PEPFAR’s commitment to mental health integration. To reach this global objective, we identified 5 key strategies that can serve as the conceptual basis for framing mental health programming as a part of PEPFAR’s current 5-year strategic plan (*27*). Each pillar represents a strategic direction that PEPFAR considers a global response priority, and initiatives laid out under each can uniquely deliver programmatic results to drive the transformative change needed for global HIV and TB programming into the future:

• Pillar 1—Health Equity for Priority Populations: Prioritize the use and collection of national mental health data to characterize potential barriers and identify potential gaps in health equity (including stigma and discrimination) that may prevent priority populations from accessing HIV and TB services.
• Pillar 2—Sustaining the Response: In collaboration with the Ministries of Health, ensure comprehensive mental health and substance use disorder services (recognized as a key element of UHC) are accessible at all PEPFAR-supported facilities. Such services include mental health screening, psychologic treatments, psychosocial support, referrals, and psychiatric medications.
• Pillar 3—Public Health Systems: Accelerate the integration of a mental health infrastructure that effectively supports the HIV and TB response into local public health systems. Focus on close partnerships with government programs, local communities, organizations, and support groups, as well as other key mental health stakeholders that may help assess health system gaps and identify opportunities for greater mental health funding within existing budget envelopes.
• Pillar 4—Transformative Partnerships: Ensure governments and implementing partners engage with the mental health community at local, national, regional, and global levels, because these key stakeholders will likely contribute additional resources and subject matter expertise needed to rapidly expand mental health programs for persons living with HIV and TB. Ensure partnerships include persons with lived experience and those aware of how mental health and substance use disorders intersect with the societal and cultural norms of the community.
• Pillar 5—Follow the Science: Aptly consider developing cost-effective programming that is supported by evidence-based interventions that address the strong association between mental health, HIV, and TB. For example, economic models suggest that for every $1.00 (US) invested in treating mental disorders, up to $5.70 is saved in economic cost and health returns (*28*). Specifically, for every dollar spent treating HIV or TB, that savings is estimated at $6.40 and $40, respectively (*29*). In addition, use the best available scientific evidence on mental health and substance use disorder interventions to guide PEPFAR’s program recommendations.

We are encouraged by the global discussion on mental health, including a recent publication co-authored by Ambassador Dr. John N. Nkengasong, US Global AIDS Coordinator, that stated, “PEPFAR intends to explore how the program’s model of care can be used to address coexisting mental health conditions among people living with HIV” (*30*). In 2021, an estimated 386,862 persons living with HIV had TB in PEPFAR-supported countries (*31*). Those persons should be considered when addressing mental health disorders as a part of routine practice because the integrated service changes will require the involvement of both the HIV and TB programs (*32*). This shifting focus toward mental health concerns creates an opportunity for mental health stakeholder engagement and advocacy. Sweetland and colleagues found a high receptivity among directors of the National Tuberculosis Programs to integrate the management of TB and mental health disorders into their policies and guidelines worldwide (*33*).

The momentum around mental health services has extended into WHO’s Comprehensive Mental Health Action Plan 2013–2030 (*14*), which, when updated in 2021, included language around universal coverage for mental health services. Member states then committed to a set of ambitious global targets, that may influence PEPFAR’s current standard health services, including aims to double the number of community-based mental health facilities as well as increase service coverage for mental health conditions by at least half by 2030 (*14*). Skeptics claim that most countries currently lack adequate resources to focus on mental health programming, but some meaningful progress may still be achieved with the resources available. For example, countries can begin initiating routine data collection and reporting of a core set of mental health indicators through their national health and social information systems, conducting mental health research informing country-specific needs, and preparing the workforce for integration of mental health services into primary health care and beyond. There is no better time than now to begin training front-line staff to help identify, manage, and refer persons with serious mental health and substance-use problems across the PEPFAR health services platform. However, what can be achieved with current resources will vary greatly. For example, countries with a significant lack of mental health subject matter expertise or training opportunities can hinder task shifting and impede programmatic progress.

Advocates agree that bold measures are crucial as the world starts an aggressive push to end the HIV and TB epidemics by 2030 (*10*), which may involve breaking down traditional programmatic silos, sharing resources, and being open to working in new ways with partners. The literature unequivocally supports the bidirectional effects of mental health and HIV and TB outcomes (*3*,*9*). Without global action and a concerted effort by national, international, and multisectoral organizations to work toward harmonizing global mental health priorities and actions, mental health problems, including substance-use disorders, will likely continue to increase the disease burden among the populations affected by HIV and TB. PEPFAR aims to reinvigorate the response to end the HIV/AIDS pandemic. Providing mental health services to persons living with both HIV and TB might improve treatment adherence and treatment outcomes, reduce community transmission, and decrease the number of deaths attributable to both diseases. Mental health, a known driver of HIV and TB treatment outcomes, stands today as a critical programmatic void in the context of PEPFAR initiatives that, if not filled, can impede decades of progress made toward the global elimination of both diseases.
